# A sustainable biorefinery to convert agricultural residues into value-added chemicals

**DOI:** 10.1186/s13068-016-0609-8

**Published:** 2016-09-17

**Authors:** Zhiguo Liu, Wei Liao, Yan Liu

**Affiliations:** Department of Biosystems and Agricultural Engineering, Michigan State University, 524 S. Shaw Ln. Room 203, East Lansing, MI 48824-1323 USA

**Keywords:** Anaerobic digestion, Animal wastes, Biorefinery, Chitin/chitosan, Electrocoagulation, Fungal fermentation

## Abstract

**Background:**

Animal wastes are of particular environmental concern due to greenhouse gases emissions, odor problem, and potential water contamination. Anaerobic digestion (AD) is an effective and widely used technology to treat them for bioenergy production. However, the sustainability of AD is compromised by two by-products of the nutrient-rich liquid digestate and the fiber-rich solid digestate. To overcome these limitations, this paper demonstrates a biorefinery concept to fully utilize animal wastes and create a new value-added route for animal waste management.

**Results:**

The studied biorefinery includes an AD, electrocoagulation (EC) treatment of the liquid digestate, and fungal conversion of the solid fiber into a fine chemical—chitin. Animal wastes were first treated by an AD to produce methane gas for energy generation to power the entire biorefinery. The resulting liquid digestate was treated by EC to reclaim water. Enzymatic hydrolysis and fungal fermentation were then applied on the cellulose-rich solid digestate to produce chitin. EC water was used as the processing water for the fungal fermentation. The results indicate that the studied biorefinery converts 1 kg dry animal wastes into 17 g fungal biomass containing 12 % of chitin (10 % of glucosamine), and generates 1.7 MJ renewable energy and 8.5 kg irrigation water.

**Conclusions:**

This study demonstrates an energy positive and freshwater-free biorefinery to simultaneously treat animal wastes and produce a fine chemical—chitin. The sustainable biorefinery concept provides a win–win solution for agricultural waste management and value-added chemical production.

**Electronic supplementary material:**

The online version of this article (doi:10.1186/s13068-016-0609-8) contains supplementary material, which is available to authorized users.

## Background

There are 450,000 animal feeding operations (AFOs) in the U.S., which produces approximately 1.3 billion wet tons (335 million dry tons) of animal wastes per year [1, 2]. Animal wastes are of particular environmental concern due to greenhouse gases emission, odor problem, and potential surface and ground water contamination. A recent trend in animal waste management is the renewed interest in using anaerobic digestion (AD) technology for energy production and carbon sequestration [3, 4]. Even though AD is an effective method for producing methane energy and reducing volatile organics, it is incompetent to sequester all carbons and remove nutrients in animal wastes. After digestion, solid digestate still has a high carbon content [5, 6], and liquid digestate contains significant amounts of nitrogen, phosphorus, and total solids [7, 8].

Many studies have been carried out to treat liquid digestate such as active carbon adsorption [9], chemical coagulation and flocculation [10], UV treatment [11] and ozone treatment [12]. Regardless good treatment performance of these methods, high-energy input and additional chemical usage make them less attractive to be commercially implemented. Meanwhile, electrocoagulation (EC) has recently been studied to treat high-strength wastewater (high solids and chemical oxygen demand) [13]. Due to its high removal efficiency and chemical-free nature, EC technology has a short retention time and avoids a secondary pollution [14]. Our previous studies have successfully established an EC treatment process that is capable of simultaneously treating AD liquid effluent and cleaning up raw biogas, and developed a tandem membrane filtration process to purify the EC treated water [15]. The relatively clean EC treated water can then be used as the processing water for cellulosic biorefinery.

As for solid digestate, treatments such as composting and incineration have been widely used [16, 17]. Besides these traditional methods, Sun et al. applied pyrolysis to convert solid digestate into biochar as adsorbent material [18]. Biological conversion processes have also been developed to use solid digestate as a viable cellulosic feedstock for bioethanol and biodiesel production [19, 20]. These studies indicate that solid digestate has much better commercial uses as a cellulosic biorefining feedstock rather than a soil amendment or a combustion fuel.

However, investigations on fully utilizing AD effluent (both solid digestate and liquid digestate) for value-added chemical production have not been reported to date. New technologies are urgently needed to realize such utilization, so that environmentally sound and economically feasible animal waste management can be achieved.

Chitin is a natural amino polysaccharide widely distributed in the animal and plant kingdom. The structure of chitin is a linear polysaccharide made up of unbranched β-(1,4)-2-acetamido-2-deoxy-d-glucopyranosyl residues which is also called N-acetyl-d-glucosamine. The structural characteristics make chitin a very attractive biopolymer that can be used as coagulating agents in wastewater treatment, plant seed coating agents in agricultural industry, and biomaterials (e.g., absorbable sutures) in biomedical industry [21, 22]. Traditionally, chitin is extracted from crustacean insects and shell fishes. Compared to the chitin from shellfishes, fungal chitin has advantages of lower level of inorganic materials, no geographic or seasonal limitations [23, 24], and better effectiveness in inducing the plant immune response (as a fertilizer) [25].

**Fig. 1:**
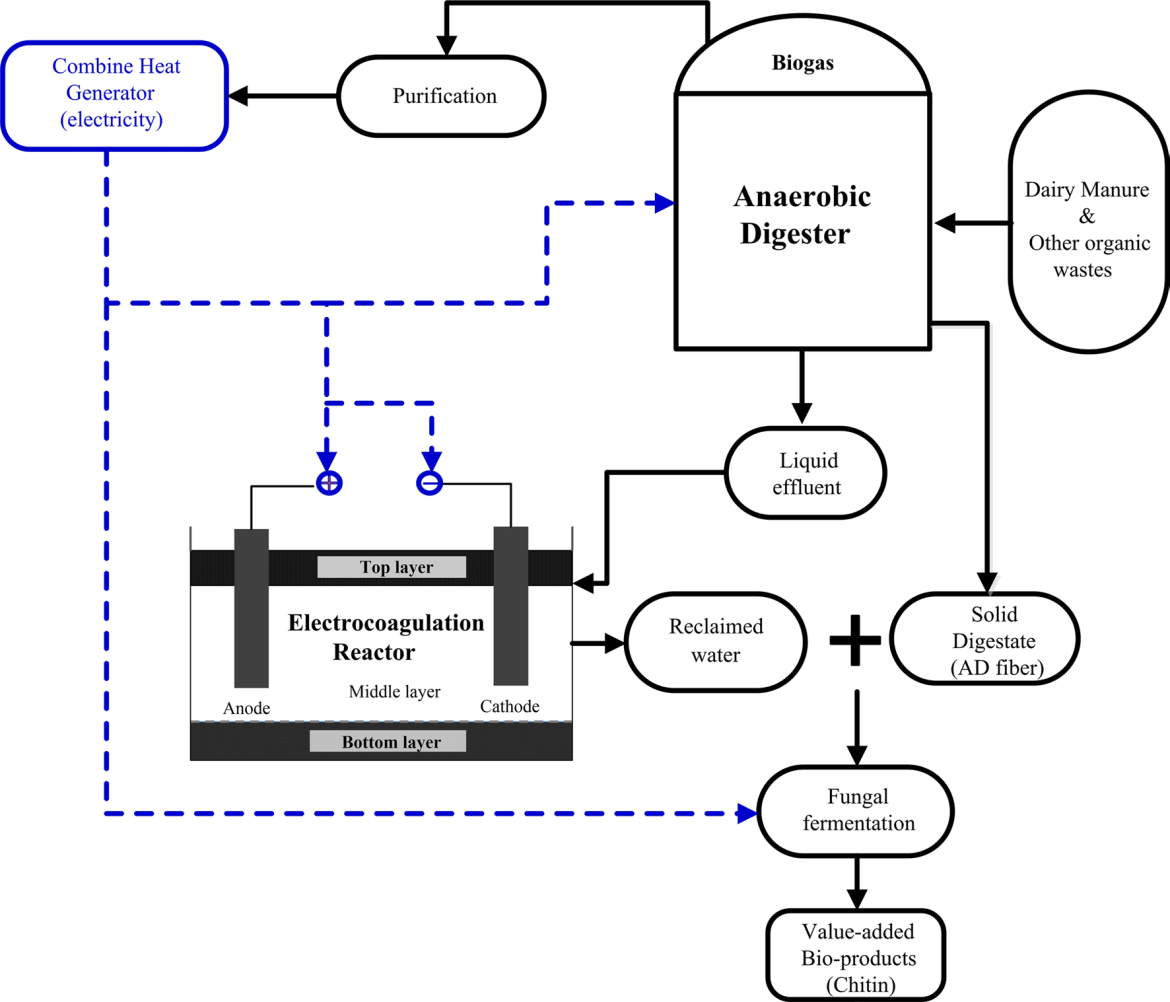
Self-sustaining biorefinery concept. *Black lines* are for mass flow; *blue lines* are for energy flow

Therefore, to convert animal wastes into a high-value chemical—chitin, this paper developed a sustainable biorefinery concept integrating AD, EC and fungal fermentation (Fig. 1). Animal wastes were first treated by an AD to produce methane gas for energy generation to power the entire biorefinery. The resulting liquid digestate was treated by EC to reclaim water. Pretreatment, enzymatic hydrolysis and fungal fermentation were then applied on the cellulose-rich solid digestate using the EC reclaimed water as the processing water to produce chitin. The studied biorefinery not only converts animal wastes into high-value added products, but also eliminates freshwater use and external power supply, which represents a promising utilization path of agricultural waste management.

## Methods

### Anaerobic digestion

Anaerobic digestion of animal wastes was carried out on a commercial anaerobic digester located at a private dairy farm (3000 cows) in Michigan (42N 46′29.51″, 85W 19′10.14″). The animal feeds of the dairy farm were alfalfa and corn silage, which are blended based on the Natural Research Council (NRC)’s standard total mixed rations (TMRs) for dairy cattle [26]. The farm uses corn straw as the bedding materials, and adopts a scrape system to collect animal feces. The digester is a completely stirred tank reactor (CSTR) operated at temperature of 40 °C and retention time of 22 days. The effective volume of the digester is 10,000 m^3^. The biogas is combusted by two 400 kW caterpillar® generators to produce electricity. Two 5.5 kW FAN® screw press separators with 2 mm screen are implemented to separate liquid and solid digestate of the AD effluent. The liquid and solid digestates were used to carry out the following EC treatment and fungal fermentation, respectively.

### EC treatment of liquid digestate

EC was conducted in a column EC reactor described in a previous study [27] with minor modifications. Current level, retention time, and working volume were set as 10A, 150 min and 3.5 L, respectively, which were determined based on COD removal of the EC (Additional file 1: Figure S1). Total solid (TS) of the liquid digestate was 2.7 %. Voltage was monitored during the EC treatment. The EC effluent was collected and centrifuged at 230*g* for 10 min to prepare EC water for the following experiments.

### Fungal fermentation of solid digestate

#### Pretreatment and enzymatic hydrolysis of solid digestate

The EC water was used as the processing water to carry out pretreatment and enzymatic hydrolysis of solid digestate. Based on the optimization (Additional file 1: Tables S1 and S2), the preferred pretreatment condition of 2 % of NaOH, 120 °C of reaction temperature, and 2 h of reaction time was selected with total solid loading fixed at 10 % (w/w). The pH of the treated slurry was adjusted to 5.5 using 30 % sulfuric acid. C-TEC3 enzyme cocktail with H-TEC (sponsored by Novozyme North America, Franklinton, NC) was then added into the slurry to release mono-sugars under the conditions of 63 h of reaction time, 50 °C of reaction temperature, and 150 rpm of shaking speed. The enzyme cocktail was prepared as: 9.10 mg cellulose (CTEC3, protein content of 218 mg mL^−1^) and 1.43 mg xylanase (HTEC3, protein content of 171 mg mL^−1^) per gram dry solid digestate. The hydrolysate was centrifuged at 7025*g* for 10 min, and the supernatant was further detoxified by Ca(OH)_2_ prior to the fermentation. The pH of the supernatant was adjusted to 10 with addition of Ca(OH)_2_ and the solution was maintained at 50 °C for 5 h with a shaking speed of 150 rpm. The Ca(OH)_2_ treated supernatant was centrifuged at 7025*g* for 10 min again. The detoxified supernatant was collected. The pH was adjusted to 6.0 before the supernatant was stocked at −20 °C for further uses. All non-specified reagents were purchased from Sigma-Aldrich®.

#### Fungal strain and fermentation process

*Rhizopus oryzae* ATCC 20344 (purchased from ATCC) was the strain used for chitin accumulation. Spores of *R. oryzae* ATCC 20344 were collected from the culture on the potato dextrose agar (PDA) medium (Sigma-Aldrich®). The spore concentration of the collected spore solution was approximately 10^7^ spores/mL. 0.5 mL of the spore solution were inoculated to 100 mL of sterilized potato dextrose broth (PDB) medium (Sigma-Aldrich®) with 8 g L^−1^ yeast extract (Acumedia^®^), and cultivated at 30 °C, 180 rpm for 36 h to prepare the seed. The detoxified solution from “Pretreatment and enzymatic hydrolysis of solid digestate” section was mixed with 3 g L^−1^ of CaCO_3_ and trace elements [28], and sterilized under 121 °C for 15 min to prepare the fermentation medium. 5 mL of the seed was inoculated to 45 mL of the fermentation medium. The fermentation was carried out at 30 °C and 180 rpm for 120 h. Samples were taken during the process to monitor kinetics of substrate consumption, growth, and product production.

### Analytical methods

Chemical oxygen demand (COD), total phosphate (TP) and total nitrogen (TN) of animal wastes, liquid digestate, and EC treated water were measured using analytical kits purchased from HACH company [13]. TS, volatile solids (VS), cellulose, hemicellulose, and lignin of animal wastes and solid digestate were analyzed using the methods developed by National Renewable Energy Laboratory (NREL) [29]. Dissolved total organic carbon (TOC) of the liquid digestate was measured by a method previously reported [13]. A Shimadzu high-performance liquid chromatography (HPLC) equipped with Aminex 87H column, micro de-ashing guard column and a refractive index detector was used to analyze the sugars and organic acids. The HPLC method was adopted from a previous study [28]. Cellulose conversion was calculated as reported [5]. Xylan conversion was calculated as ((Volume of enzymatic hydrolysate) (L) * (Xylose concentration) (g L^−1^))/((Weight of solid digestate used for pretreatment) (g) * (Total solid content) (% w/w) * (Xylan content) (% w/w) * 1.136) * 100. Chitin/chitosan were extracted from the collected fungal biomass [30, 31], and glucosamine content was also measured [32].

### Statistical analysis

General linear model (GLM) analysis using the Statistical Analysis System program 9.3 (SAS Institute, Inc. Cary, NC) was conducted to select the preferred condition for pretreatment. Temperature, alkali loading, and reaction time were the parameters. Total sugar concentration (glucose + xylose) was the response. Analysis of variance (ANOVA) was used to interpret the data and draw conclusions.

## Results and discussion

### Anaerobic digestion

The characteristics of animal wastes (AD feedstock) were analyzed and summarized in Table 1. High concentrations of COD, TN and TP in the animal wastes provide good nutritious sources to support growth of anaerobic microbes. 454 metric tons of the wet animal wastes are fed daily into the digester. Under 22 days of hydraulic retention time (HRT) and 40 °C of culture temperature, the AD generates 8495 m^3^ biogas per day with a methane content of 60 % (v/v), and produces 40 metric tons wet solid digestate and 397 metric tons liquid digestate per day. The energy demand to maintain the temperature of the AD and power accessory equipment is 5760 MJ/day.

**Table 1:** Characteristics of animal wastes and performance of the commercial CSTR digester

Characteristics of animal wastes (AD feedstock)	Value^a^
Total solids (%,TS)	7.97 ± 0.45
Volatile solids (%, VS)	78.61 ± 1.31
COD (mg L^−1^)	93,450 ± 2474
TP (mg L^−1^)	2423 ± 49.33
TN (mg L^−1^)	3673 ± 110.2

Digester performance	Value
Operating temperature (°C)	40
HRT (days)	22
Biogas production (m^3^ day^−1^)	8495
Methane composition (%)	60
Animal wastes feeding the AD (wet tons day^−1^)	454
Solid digestate generated (wet tons day^−1^)	40
Liquid digestate generated (tons day^−1^)	397
Average energy demand for the AD operation (MJ day^−1^)	5760
Methane production per COD in the AD feedstock (m^3^ kg^−1^)	0.13

^a^ Data are average of three replicates with standard deviation

As aforementioned, AD is a natural and biological process good at confining organic wastes and producing renewable energy, though, it has limitations on completely degrading fiber and removing nutrients in agricultural wastes [5, 6]. A large portion of cellulose, hemicellulose and lignin remained in the solid digestate (Table 2), and nutrients (P and N) in inorganic form exist in both liquid and solid digestates (Table 3). To improve the efficiency of animal waste utilization, it is in great need of new approaches to convert these remaining compounds into value-added chemicals. EC and fungal fermentation were adopted by this study to produce chitin from the digestates.

**Table 2:** Characteristics of solid digestate and hydrolysate as well as cellulose and xylan conversion during the pretreatment and enzymatic hydrolysis

Characteristics of solid digestate	Value^a^
Total solids (% TS)	26.27 ± 1.11
Volatile solids (% VS)	87.70 ± 0.44
Cellulose (% TS)	20.56 ± 0.21
Xylan (% TS)	11.77 ± 0.39
Lignin (% TS)	33.05 ± 0.23

Sugar and acid concentrations of hydrolysate^b^	Value^a^
Glucose (g L^−1^)	15.78 ± 0.36
Xylose (g L^−1^)	11.49 ± 0.15
Acetate (g L^−1^)	2.23 ± 0.10

Cellulose and xylan conversion	Value^a^
Cellulose conversion (%)	64.34 ± 2.28
Xylan conversion (%)	78.18 ± 2.77

^a^ Data are average of three replicates with standard deviation

^b^ The concentrations were for the hydrolysate after pretreatment, enzymatic hydrolysis and detoxification

**Table 3:** Characteristics of liquid digestate and EC water and performance of EC treatment

	Characteristics	Value
Liquid digestate^a^	Total solids (% TS)	2.64 ± 0.03
	COD (mg L^−1^)	9490 ± 14.1
	TP (mg L^−1^)	120 ± 0.0
	TN (mg L^−1^)	1495 ± 43.84
	TOC (mg L^−1^)	4284 ± 326
EC water^a^	Total solids (% TS)	0.78 ± 0.11
	COD (mg L^−1^)	1706.2 ± 19.4
	TP (mg L^−1^)	9.25 ± 0.35
	TN (mg L^−1^)	997.5 ± 31.82
Removal efficiency	TS removal (%)	70.5
	COD removal (%)	82.0
	TP removal (%)	92.3
	TN removal (%)	33.3

^a^ Data are average of three replicates with standard deviation

### Electrocoagulation of the liquid digestate

It has been tested that the liquid digestate with a high COD concentration is not amendable for fungal fermentation of chitin accumulation (data not shown). The liquid digestate must be treated prior to use as the processing water for the fermentation. EC as a non-membrane technology has advantages of high TS and COD removal efficiencies and dual-function of biogas clean-up and water reclamation [13], so that EC was adopted to treat the liquid digestate in this study. Table 3 shows the characteristics of liquid digestate and EC water as well as the performance efficiency of the EC treatment. Removal of TS, COD, TP, and TN during the EC were 70.5, 82, 92.3 and 33.3 %, respectively. Compared to the removal of TS, COD, and TP, EC has lower efficiency on TN removal. It has been reported that EC is highly efficient in removing solid-dependent nutrients—TS, TP and COD [14], while it is incompetent in removing highly soluble compounds from solution such as ammonium ion (the main form of nitrogen in the liquid digestate) [13, 27]. Nevertheless, high level of nitrogen is favorable for fungal biomass growth and chitin synthesis, while limits production of other nontarget metabolites such as lactic acid and fumaric acid [33–35]. Therefore, using EC water with high nitrogen content as the processing water could be beneficial for *R. oryzae* culture to limit lactic acid production and accumulate more chitin.

Energy consumption is the main concern for the EC process. Electricity used during the EC process was monitored. The voltage was kept stable at 16 ± 4 V in the first 120 min, and increased to 30 V in the last 30 min of the process when the EC water turned into a relatively clear solution. According to the electrocoagulation principle, colloidal condition formed by charged (mostly negatively) particles has to be primarily broken to trigger massive precipitation [14, 36]. Such solid precipitation leads to increase of electronic resistance, and subsequently results in the rapid climbing of voltage. The total energy consumption of the EC was 446 kJ/L liquid digestate.

### Fungal conversion of solid digestate into chitin using the EC water as the processing water

#### Pretreatment and enzymatic hydrolysis of solid digestate using the EC water as the processing water

The solid digestate has relatively high contents of cellulose (21 % TS) and xylan (12 % TS), which provides a good carbohydrate source. A three-step process of pretreatment, enzymatic hydrolysis and detoxification was applied on the solid digestate to convert cellulose and hemicellulose into mono-sugars for *R. oryzae* fermentation. The EC water was used as the processing water. The hydrolysate after the three-step process contained 16 g L^−1^ glucose, 11 g L^−1^ xylose, and 2 g L^−1^ acetate. The cellulose and xylan conversion were 64 and 78 %, respectively, which are well aligned with a previous study [5]. The results also demonstrate that the EC water had no negative impacts on pretreatment, enzymatic hydrolysis or detoxification of the solid digestate.

#### Fungal fermentation on the hydrolysate to produce chitin

**Fig. 2:**
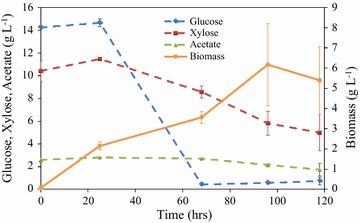
Kinetics of fungal growth and substrate utilization. Data are average of three replicates with standard deviation

Fungal fermentation was carried out using the hydrolysate as the medium. The kinetic data demonstrate that *R. oryzae* can utilize glucose and xylose in the hydrolysate to accumulate biomass and produce chitin (Fig. 2). However, the consumption of glucose and xylose was observed in a tandem pattern where xylose utilization was after near-complete consumption of glucose. In addition, glucose was consumed much faster than xylose, which verified that *R. oryzae* prefers glucose to xylose as a carbon source [37]. Acetate was not significantly consumed during the fermentation, indicating that acetate is not a carbon source for *R. oryzae*. It is also interesting to observe that there was minimum lactate accumulation during the fermentation on the hydrolysate. It has been reported that lactate metabolism of *R. oryzae* is significantly influenced by the nitrogen content in the medium [34]. High level of nitrogen tends to be more favorable for cell growth and chitin synthesis than lactate accumulation. The EC water as the processing water contains 998 mg L^−1^ of total nitrogen, which most likely influenced the fermentation for biomass accumulation and no lactate production. At the end of the exponential growth phase (96 h), the biomass reached the maximum concentration of 6.17 g L^−1^. The corresponding biomass yield was 33 % with respect to the amount of consumed glucose and xylose. However, even though xylose has been consumed by *R. oryzae*, there was still 5.81 g L^−1^ of xylose left in the broth at the end of the exponential growth phase. The xylose utilization efficiency was only 44 %. Improving xylose utilization of *R. oryzae* is critical to improve carbon utilization efficiency, and is currently under investigation.

**Fig. 3:**
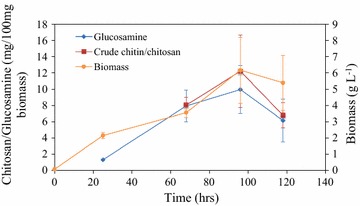
Kinetics of chitin/chitosan and glucosamine accumulation. Data are average of three replicates with standard deviation

Correspondingly, relationship between chitin/chitosan, glucosamine and biomass during the fermentation was also delineated (Fig. 3). Similar to the growth kinetics, chitin/chitosan and glucosamine all peaked at 96 h, which is consistent with the reported observation that extractable chitin content maximized at the end of exponential phase [23]. The maximum concentrations of chitin/chitosan and glucosamine were 0.75, and 0.50 g L^−1^, respectively. The yields of chitin/chitosan and glucosamine were 4.10 and 2.73 % based on the amount of consumed glucose and xylose.

Several fungal strains such as *Aspergillus niger*, *Mucor rouxii*, and *Candida albicans* have been studied to produce chitin/chitosan on different feedstock (Table 4). Among them, *R. oryzae* is the one that demonstrates better performance on chitin accumulation. Higher chitin content and yield of *R. oryzae* were observed in previous studies (Table 5). However, most of them used pure sugar or starch as the feedstock. There were only a few studies partially using agricultural residues as feedstock for chitin production [33, 34, 38]. This study is the first report that uses animal wastes as the sole carbon source to culture *R. oryzae* and accumulate chitin.

**Table 4:** Partial fungal chitin/chitosan production summary

Origin strain	Feedstock	Fermentation time (days)	Chitin/chitosan content	Reference
*Rhizopus oryzae ATCC 20344*	100 % AD fiber with treated AD effluent	3	12.2	This study
*Aspergillus niger*	Yeast, peptone and dextrose broth	15	11.1^a^	[23]
*Mucor rouxii*	Yeast, peptone and dextrose broth	21	20.13^a^	[23]
*Rhizopus oryzae MTCC 262*	Deproteinized whey	3	11.9	[38]
*Rhizopus oryzae NRRL 395*	Steamed rice	3	20^b^	[45]
*Rhizopus oryzae 0602*	Glucose, peptone, yeast extract, etc.	4	4.91	[46]
*Rhizopus oryzae 0263*	Glucose, peptone, yeast extract, etc.	4	4.43	[46]
*Cunninghamella echinulata*	Glucose, peptone, yeast extract, etc.	4	7.14	[46]
*Aspergillus niger TISTR3245*	PDB	16	11	[47]
*Rhizopus oryzae TISTR3189*	PDB	6	14	[47]
*Zygosaccharomyces rouxii TISTR5058*	PDB	2	3.6	[47]
*Candida albicans TISTR5239*	PDB	2	4.4	[47]
*Rhizopus oryzae YPF*-*61A*	Glucose	6	7.5	[48]
*Rhizopus oryzae NRRL 395*	100 % potato hydrolysate	3	25	[34]
*Rhizopus oryzae ATCC 20344*	50 % manure liquid with 20 g/L glucose	2	21	[33]

^a^ Data shown are glucosamine content

^b^ Data shown is chitin/chitosan content only in mycelia

**Table 5:** Energy balance of the self-sustaining biorefinery

Energy balance^a^	AD	EC process	Fungal fermentation ^b^
Energy input (MJ/kg dry feedstock)	−0.16^c^	−1.47^d^	−3.63^e^
Energy output (MJ/kg dry feedstock)	6.95^f^	0	0
Net energy (MJ/kg dry feedstock)	6.79	−1.47	−3.63
Overall net energy (MJ/kg dry feedstock)	1.69		

All inputs are negative, and all outputs are positive

^a^ Data were calculated and adjusted based on 1 kg dry animal wastes

^b^ The fungal fermentation includes unit operations of pretreatment, enzymatic hydrolysis and fungal fermentation

^c^ The energy input for the AD unit includes both heat and electricity

^d^ The energy input for the EC unit is 446.65 kJ/L liquid digestate

^e^ The energy input for pretreatment, enzymatic hydrolysis, fungal fermentation and post-processing is 1.25 MJ/L fermentation broth (unpublished data)

^f^ The energy output of the AD is the methane energy. Low heating value of methane of 50 kJ/g methane was used for the calculation

### Mass and energy balance analysis

**Fig. 4:**
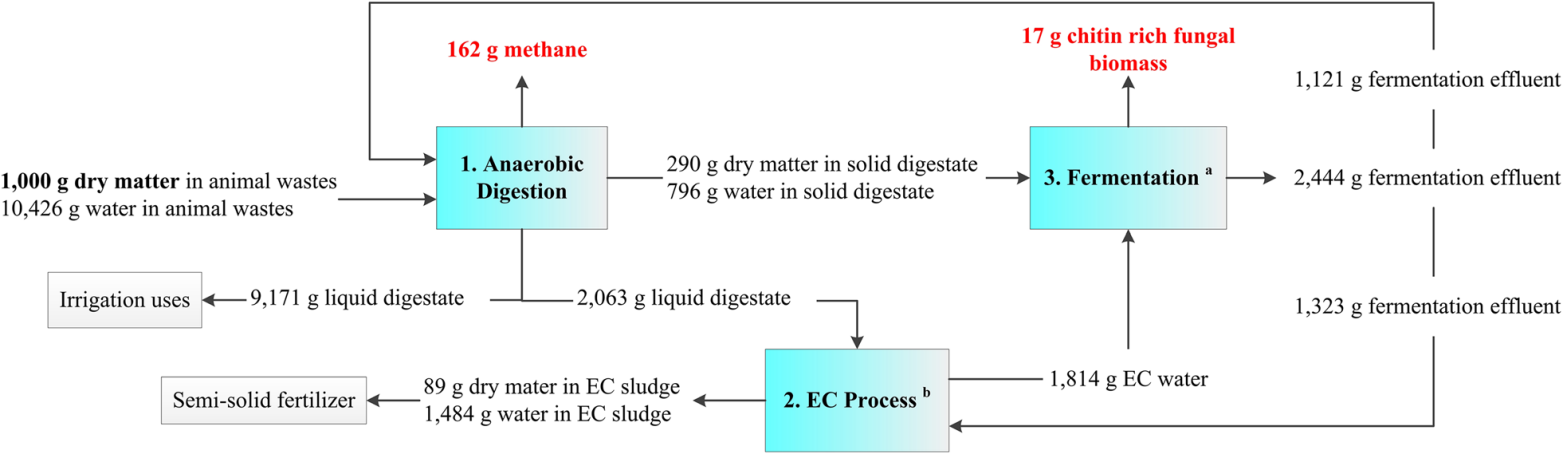
Mass balance of the self-sustaining biorefinery. The overall mass balance analysis was based on 1000 g dry animal wastes. *a* The mass balance for fungal fermentation was calculated based on 50 mL flask data. *b* The EC process used the mixture of fermentation effluent and liquid digestate to generate the EC water for the fermentation use

A mass and energy balance was conducted to evaluate the system performance (Fig. 4). The AD generated 162 g methane, 290 g solid digestate, and 11,234 g liquid digestate per kg dry animal wastes (Fig. 4). A portion of the liquid digestate (2063 g per kg dry animal wastes) mixed with 1323 g fermentation effluent per kg dry animal wastes was treated by EC to prepare the EC water for fermentation use. The EC sludge (1573 g per kg dry animal wastes) rich in phosphorus can be used as a fertilizer. The fungal fermentation on the hydrolysate of the solid digestate generated 17 g fungal biomass per kg dry animal wastes containing 12 % of chitin and 10 % of glucosamine. The water was completely self-sustained, and the freshwater was not needed. In addition, the EC water can cover the processing water for the fungal fermentation. A large demand of freshwater is one of the major challenges for fermentation processes of value-added chemical production [39–42]. Applying wastewater as processing water is becoming favorable to make the bioprocesses more sustainable [43, 44]. The results in this study demonstrate that combining AD and EC can generate the processing water to satisfy the demand of the fungal fermentation for value-added chitin production. Besides the EC water used as the processing water, there was an extra amount of liquid digestate (9171 g/kg dry animal wastes) rich in nitrogen and phosphorus, which can be used as a liquid fertilizer.

Energy balance also demonstrates that integrating AD with EC and fungal fermentation leads to an energy positive biorefining process (Table 5). AD as a powerhouse in the system generated 6.95 MJ energy per kg animal wastes. EC and fungal fermentation (with pretreatment and hydrolysis) consumed 1.47 and 3.63 MJ per kg animal wastes, respectively, to satisfy the demands of water treatment and fermentation process to convert 290 g of solid digestate into 17 g of chitin/chitosan. A positive net energy output of 1.69 MJ per kg animal wastes was achieved by the studied biorefining concept.

## Conclusion

The biorefinery system can produce 17 g fungal biomass with 12 % chitin from 1 kg dry animal wastes. The mass and energy balance analysis concludes that the biorefinery is an energy neutral and freshwater-free biorefining system with a net energy and water outputs of 1.69 MJ/kg dry animal wastes and 8.5 kg/kg dry animal wastes, respectively. Correspondingly, the self-sustaining concept that synergistically integrates AD, EC, and fungal fermentation to convert agricultural wastes into value-added product is concluded. The concept provides a win–win solution for agricultural waste management and biorefining of value-added chemical production.

## Additional file

10.1186/s13068-016-0609-8 Additional figure and tables. Additional information is available at Biotechnology for Biofuels journal’s website.

## Abbreviations

AD: anaerobic digestion
HRT: hydraulic retention time
EC: electrocoagulation
COD: chemical oxygen demand
TS: total solids
VS: volatile solids
TP: total phosphorus
TN: total nitrogen
