# Supplementary material for: A sustainable biorefinery to convert agricultural residues into value-added chemicals
Source: Biotechnol Biofuels. 2016 Sep 17;9:197. doi: 10.1186/s13068-016-0609-8 (PMC5027126; doi:10.1186/s13068-016-0609-8)
Supplement: Supplementary file 1 — 10.1186/s13068-016-0609-8 Additional figure and tables. Additional information is available at Biotechnology for Biofuels journal’s website. [file 13068_2016_609_MOESM1_ESM.docx]

**A Sustainable Biorefinery to Convert Agricultural Residues into Value-Added Chemicals**

Supplemental Material

Zhiguo Liu, Wei Liao, Yan (Susie) Liu*

Biosystems and Agricultural Engineering, Michigan State University, East Lansing, MI 48824

Figure S1. COD during EC treatment of AD liquid effluent*

*: Data are the average of two replicates.

Table S1. Complete random design (CRD) of pretreatment conditions using EC water

|  | Levels | Specifications |
| --- | --- | --- |
| Temperature | 2 | 120 °C, 130 °C |
| NaOH conc. | 2 | 2%, 3% |
| Time | 2 | 2hrs, 3hrs |

Table S2. ANOVA analysis summary

| Source | DF | Sum of Squares | Mean Square | F Value | Pr > F |
| --- | --- | --- | --- | --- | --- |
| Model | 7 | 48.26 | 6.89428571 | 1.85 | 0.2032 |
| Error | 8 | 29.78 | 3.7225 |  |  |
| Corrected Total | 15 | 78.04 |  |  |  |

Table S3. Type III error analysis summary

| Source | DF | Type III SS | Mean Square | F Value | Pr > F |
| --- | --- | --- | --- | --- | --- |
| Temp | 1 | 12.96 | 12.96 | 3.48 | 0.099 |
| Time | 1 | 19.8025 | 19.8025 | 5.32 | 0.05 |
| Temp*Time | 1 | 1.8225 | 1.8225 | 0.49 | 0.5039 |
| NaOH conc. | 1 | 8.7025 | 8.7025 | 2.34 | 0.1648 |
| Temp*NaOH Conc. | 1 | 0.2025 | 0.2025 | 0.05 | 0.8214 |
| Time*NaOH Conc. | 1 | 4.41 | 4.41 | 1.18 | 0.3081 |
| Temp*Time*NaOH Conc. | 1 | 0.36 | 0.36 | 0.1 | 0.7638 |
